# The HSP90 Inhibitor Ganetespib Radiosensitizes Human Lung Adenocarcinoma Cells

**DOI:** 10.3390/cancers7020814

**Published:** 2015-05-22

**Authors:** Roberto Gomez-Casal, Chitralekha Bhattacharya, Michael W. Epperly, Per H. Basse, Hong Wang, Xinhui Wang, David A. Proia, Joel S. Greenberger, Mark A. Socinski, Vera Levina

**Affiliations:** 1 The University of Pittsburgh Cancer Institute, Pittsburgh, PA 15213, USA; E-Mails: gomezcasalr@upmc.edu (R.G.-C); bhattacharyac@upmc.edu (C.B.); EPPERLY@pitt.edu (M.W.E.); basse@imap.pitt.edu (P.H.B.); how8@pitt.edu (H.W.); greenbergerjs@UPMC.EDU (J.S.G.); socinskima@upmc.edu (M.A.S.); 2 Department of Medicine, The University of Pittsburgh, Pittsburgh, PA 15213, USA; 3 Department of Radiation Oncology, The University of Pittsburgh, Pittsburgh, PA 15213, USA; 4 Department of Immunology, The University of Pittsburgh, Pittsburgh, PA 15213, USA; 5 Department of Biostatistics, The University of Pittsburgh, Pittsburgh, PA 15213, USA; 6 Harvard Medical School, Harvard University, 25 Shattuck Street, Boston, MA 02115, USA; E-Mail: xwang30@mgh.harvard.edu; 7 Synta Pharmaceuticals Corp., 45 Hartwell Avenue, Lexington, MA 02421, USA; E-Mail: roia@syntapharma.com

**Keywords:** HSP90 inhibitor ganetespib, lung adenocarcinoma, xenografted tumors, ionizing radiation, senescence, apoptosis, cell cycle, DNA repair

## Abstract

The molecular chaperone HSP90 is involved in stabilization and function of multiple client proteins, many of which represent important oncogenic drivers in NSCLC. Utilization of HSP90 inhibitors as radiosensitizing agents is a promising approach. The antitumor activity of ganetespib, HSP90 inhibitor, was evaluated in human lung adenocarcinoma (AC) cells for its ability to potentiate the effects of IR treatment in both *in vitro* and *in vivo*. The cytotoxic effects of ganetespib included; G2/M cell cycle arrest, inhibition of DNA repair, apoptosis induction, and promotion of senescence. All of these antitumor effects were both concentration- and time-dependent. Both pretreatment and post-radiation treatment with ganetespib at low nanomolar concentrations induced radiosensitization in lung AC cells *in vitro*. Ganetespib may impart radiosensitization through multiple mechanisms: such as down regulation of the PI3K/Akt pathway; diminished DNA repair capacity and promotion of cellular senescence. *In vivo*, ganetespib reduced growth of T2821 tumor xenografts in mice and sensitized tumors to IR. Tumor irradiation led to dramatic upregulation of β-catenin expression in tumor tissues, an effect that was mitigated in T2821 xenografts when ganetespib was combined with IR treatments. These data highlight the promise of combining ganetespib with IR therapies in the treatment of AC lung tumors.

## 1. Introduction

Lung cancer is responsible for one-third of all cancer deaths. NSCLC comprises 85% of all lung cancer cases, with adenocarcinoma (AC) representing the major histological subtype of NSCLC [1]. Despite decades of research, systemic therapies fail to cure most lung cancers [2,3,4,5]. Currently, IR therapy is an integral component of the treatment for NSCLC [3,6,7]. However, intrinsic or acquired resistance is considered to be the main limiting factor for the efficacy of IR in long-term treatment of lung cancer [7,8]. AC sensitivity to IR depends on the oncogenic genotype which is present. Tumors bearing epidermal growth factor receptor (EGFR) mutations, or those expressing the echinoderm microtubule-associated protein-like 4 gene fused to the anaplastic lymphoma kinase (EML4-ALK) gene, are largely radiosensitive. The KRAS mutant tumors tend to be resistant to IR [9,10,11,12,13]. In the clinical setting, IR therapy is routinely ordered for patients without determination of the dominant oncogenic mutations that are present [12].

Experimental data has identified the mechanisms involved in repairing the DNA damage induced by IR and chemotherapy in the radioresistant genotypes [14,15,16,17,18,19,20]. Radiosensitizing agents, inhibitors of DNA repair, have been under consideration as therapy for decades [21]. However, little progress has been made as the radiosensitizing agents tested are not tumor specific and radiosensitize both normal and tumorigenic tissues [19]. Efforts to improve the efficacy of IR therapies using such recent technologies as intensity-modulated radiation therapy (IMRT) and gamma knife (stereotactic radiosurgery) techniques have greatly reduced the damage to normal tissues. However, delivery of high-dose IR is still limited due to damage to surrounding normal tissues [22,23,24,25]. The cellular heterogeneity present within lung tumors also presents a challenge for NSCLC therapies [26]. Sensitivity to IR-therapy in NSCLC has been determined by monitoring the transition of epithelial tissues to mesenchymal tissues (EMT) [27]. We have determined that IR- resistant residual NSCLC cells express a complex phenotype combining cancer stem cells (CSCs), as well as EMT markers [28]. Radiosensitizing therapeutics which can eliminate these IR-resistant cells could have most profound impact on the efficacy of NSCLC therapies.

The abundantly expressed molecular chaperone HSP90 plays a critical protective role in folding, stabilizing, and aggregation for the hundreds of known HSP90 client proteins [29,30]. The basic mechanisms of HSP90-induced protein folding involves conformational switches between open and closed conformations that requires ATP hydrolysis [31,32]. ATP competitive inhibitors of HSP90 disrupt the chaperone cycle, resulting in the destabilization and degradation of HSP90 clients [33,34,35,36,37]. Thus, HSP90 is an attractive therapeutic target since targeted inhibition of the chaperone can impact such a large and diverse group of protein kinases, transcription factors, and E3 ligases. Many of these clients are validated oncogenic drivers of lung adenocarcinoma including mutant EGFR [38], mutant BRAF [39], wild-type and mutant HER2 [40,41], and the EML4-ALK translocation product [42]. A surprising and common finding with most HSP90 inhibitors is their selectivity for certain tumor cells and not other cells. With few exceptions *in vivo*, most cancer cells are more sensitive to HSP90 inhibition than non-transformed cells and non-toxic doses demonstrate anti-cancer activity. In animals and humans, HSP90 inhibitors consistently accumulate in tumors whereas they are rapidly cleared from plasma and do not appear to enter most tissues [43,44,45].

Ganetespib (Synta Pharmaceuticals Corp., Lexington, MA, USA) is a new potent inhibitor of HSP90 with favorable pharmacologic and safety characteristics over the first generation of HSP90 inhibitor compounds [46,47]. Ganetespib is structurally distinct, relatively hydrophobic, and considerably smaller in size to the prototypical ansamycin class of HSP90 inhibitors. Ganetespib readily penetrates, distributes and is retained throughout solid tumors in *in vivo* models [37,47]. Ganetespib has shown preclinical activity against NSCLC models, including those driven by mutant EGFR, rearranged ALK, and/or mutant KRAS [6,48,49], and has been shown to potentiate the effect of taxanes and PI3K/mTOR inhibitor BEZ235 in NSCLC models [48,49]. In clinical trials, ganetespib monotherapy has a manageable side effect profile, as well as promising clinical activity in heavily pretreated patients with advanced NSCLCs, most notably in patients with tumors harboring ALK gene rearrangements [50].

It is reasonable to suggest that pharmacological blockade of HSP90 might produce multifaceted effects on various cell signaling pathways of cancer cells, including the inhibition prosurvival oncogenic signals in IR- and drug resistant cancers [51,52]. Studies have shown that ganetespib had a radiosensitizing effect in preclinical colorectal cancer model systems [53] but has not been explored in NSCLC. Here we examine the radiosensitizing effects of ganetespib on human lung AC cells with differing genetic backgrounds, including KRAS mutant A549 cells, EGFR mutant primary T2851 cells, and primary T2821 cells that express wild type (wt) KRAS and EGFR.

## 2. Results and Discussion

### 2.1. Growth Inhibitory Effect of Ganetespib on Human Lung Adenocarcinoma Cells

**Figure 1:**
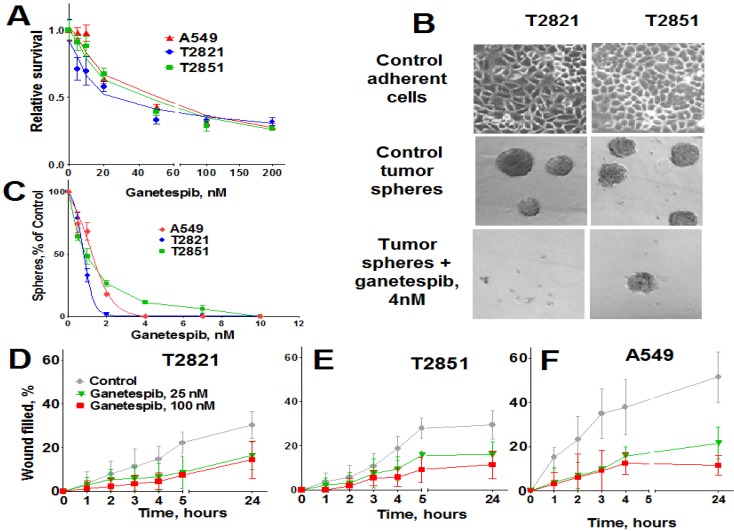
Ganetespib inhibits the proliferation of human lung AC cells growing as adherent cultures and as tumor spheres and reduces AC cells migration. (**A**). Tumor cells were grown in the presence of ganetespib (0–1000 nM) and the cellular viability was assessed after 72 h using a MTT assay. (**B**). Images of T2821 and T2851 cells cultured in adherent condition (upper panel) are shown. Images of tumor spheres formed from untreated T2821 and T2851 cells (medium panel) and cells treated with 4 nM of ganetespib (lower panel) are presented. (**C**). Ganetespib inhibits lung tumor sphere formation. Cells were grown in ultra low attachment plates in serum free media and grown in the presence of ganetespib (0–100 nM) for 7 days and then the tumor spheres were counted. Results are presented as % of control. (**D**)–(**F**). Ganetespib reduces the motility of lung AC cells. The migratory rates were determined by measuring wound width as a function of time. Data are expressed as the mean ± SD of three–six experiments.

To assess the anticancer activity of ganetespib on lung cancer cells, A549, T2821 and T2851 AC cell lines were initially cultured in the presence of graded concentrations of ganetespib for 72 h. All three AC cell lines were sensitive to the antiproliferative effects of ganetespib, with the T2821 cell line showing the greatest sensitivity (IC_50_, 21.2 ± 0.9 nM), and with lower sensitivities detected in T2851 (IC50, 43.4 ± 1.5 nM) and A549 (IC_50_, 49.9 ± 1.9 nM) cell lines (Figure 1A).

3-D tumor sphere formation is frequently used to evaluate the clonogenicity of tumor cells and to measure the growth of putative cancer stem cells (CSCs) under serum-free and ultra-low attachment conditions [54,55,56]. Ganetespib dramatically reduced tumor sphere formation in each of the cell lines (Figure 1B), with T2821 cells again showing the greatest sensitivity to treatment (IC_50_, ~0.9 nM/IC100, ~4 nM) versus (IC50, ~1.4 nM/IC100, ~4 nM) for A549 cells and (IC_50_, ~1.2 nM/IC100, ~10 nM) for cell line T2851. As shown in Figure 1C, a 4 nM concentration of ganetespib was sufficient to block sphere formation in T2821 cells; while 10 nM was required to completely abrogate this effect in T2851 cells (data not shown).

### 2.2. Ganetespib Inhibits Lung Adenocarcinoma Cell Migration

High migratory capacity is a key characteristic of metastatic tumor cells [57]. The effect of ganetespib exposure on the migration of A549, T2821 and T2851 cell lines was measured using a scratch-wound assay. A549 cells demonstrated higher migratory potential than the primary T2821 and T2851 lung tumor cells (Figure 1D,E). Ganetespib significantly reduced AC cell migration rates, in all cell lines as compared to untreated cell, suggesting that ganetespib treatment robustly inhibited cell migrations.

### 2.3. Ganetespib Induces Apoptosis, Growth Arrest, and Senescence in Lung Adenocarcinoma Cells

**Figure 2:**
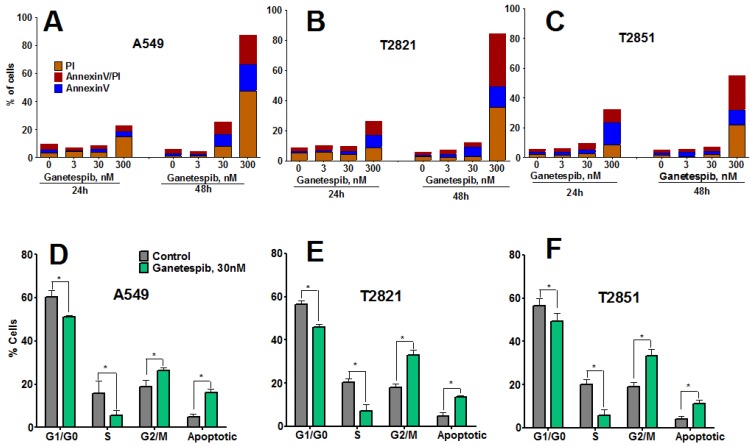
Ganetespib induces apoptosis and modulates cell cycle progression in lung AC cells. (**A**)–(**C**). Tumor cells were incubated with ganetespib (0–300 nM) for 24 or 48 h and then cells were harvested, stained with Annexin V-Alexa488/PI and measured by flow cytometry. Values are the mean of three independent experiments. (**D**)–(**F**). Cells were treated with ganetespib (0, 30 nM) for 48 h, fixed and stained with PI and the cell cycle distribution was measured by flow cytometry. Data shown are the mean ± SD of three experiments.

To determine whether growth inhibition by ganetespib cells was due to induction of apoptosis, cells were treated with graded concentrations of ganetespib for 0, 6, 24 and 48 h and apoptosis assessed by Annexin-V Alexa Fluor 488/PI staining and flow cytometry. Apoptosis was not detected in cells at the 6 h time point (data not shown), nor in cells treated with ganetespib at the lowest concentrations of 3 nM (Figure 2A–C). 

Higher concentrations of ganetespib (30, 300 nM) induced apoptosis, which was determined to be both concentration- and time-dependent. The degree of early apoptotic and late apoptotic changes and necrotic cell death differed between the cell lines, with T2851 cells showing the lowest effect as compared to the T2821 and A549 cell lines (Figure 2).To assess potential cell cycle effects in the AC cells, cells were treated with 30 nM ganetespib, for 48 h (Figure 2D,E). Cell cycle analysis revealed G2 accumulation, which was associated with a concomitant loss of S phase and decreased G1 fraction, in each of the three cell lines.

**Figure 3:**
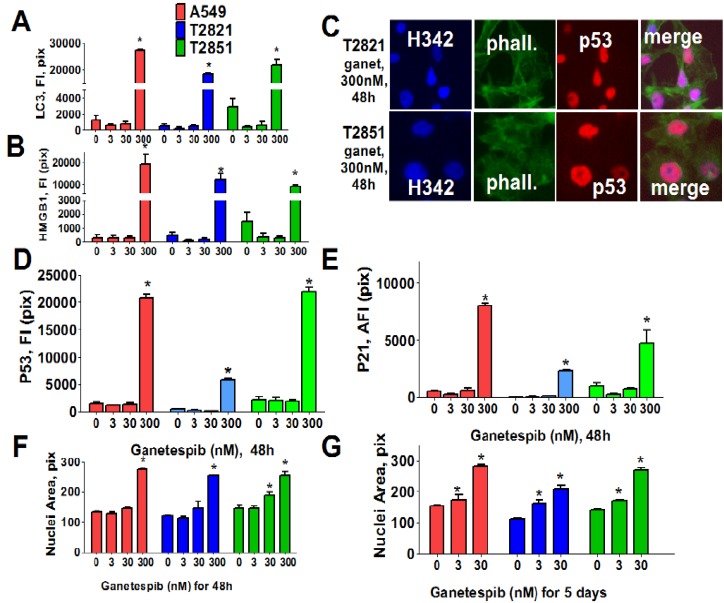
Ganetespib induces senescence in lung AC cells. Tumor cells were treated with ganetespib (0–300 nM) for 48 h and stained with primary antibodies against LC3 I/II, HMGB1, p21, p53 and F-actin (phalloidin). Each point presents the average intensities (pixels) estimated for 3000 cells. (**A**), (**B**). Analysis of autophagy associated proteins, LC3 I/II and HMGB1, in lung AC cells treated with ganetespib (0–300 nM). The total average fluorescence intensities of LC3 I/II (**A**) and HMGB1 (**B**) in cells are shown. (**C**)–(**E**). Ganetespib up regulates expressions of the senescence-associated markers (p16 and p21) in lung AC cells. (**C**). The representative images of T2821 and T2851 cells stained for p21 and for F-actin are shown. The average fluorescence intensities of nuclear p53 (**D**), and p21 (**E**) in the ganetespib-treated cells are presented. (**F**), (**G**). Ganetespib treatment leads to time-dependent and concentration-dependent increase in the sizes of nuclei in AC cells. The average nuclear areas (pix) of cells treated with ganetespib for 2 days (**F**) and for 5 days (**G**) are shown. Cells were stained with Hoechst 33342, and analyzed by a Cellomics ArrayScan HCS Reader.

These data suggested that cycle arrest may lead to activation of autophagy or senescence in ganetespib-treated cells. To examine this possibility, we next examined two markers of autophagy: Expression of light chain 3 (LC3) [58] and distribution of high mobility group 1 (HMGB1) protein [59]. Cells were cultured in increasing concentrations of ganetespib for 48 h (Figure 3) and both LC3 and HMGB1 proteins were found to be significantly up-regulated in cells treated with high dose ganetespib (300 nM) (Figure 3A,B). Nuclear localization of HMGB1 protein was suggestive of an apoptotic response rather than a macro autophagy event.

Since these effects were absent in cells treated with low concentrations of ganetespib, we next examined the role of cellular senescence in growth inhibitory phenotype induction by ganetespib treatment. To do this, multiple morphological and molecular markers of senescence were evaluated, including nuclear size change, the formation of senescence-associated foci (SAHF) [60], and expression of the cyclin-dependent kinase (CDK) inhibitors (p21, p16) as well as p53 and p27 expression. Ganetespib treatment led to an accumulation of p21 and p53, but not p16, in the nucleus of AC cells (Figure 3C–E). Correlating with this upregulation, increased p27 expression and the formation of SAHF was observed in ganetespib-treated cells (data not shown). Nuclear sizes were significantly increased in AC cells treated with 300 nM ganetespib for 48 h (Figure 3F). Extending the duration of treatment with lower concentrations of ganetespib also resulted in enlarged cell nuclei (Figure 3G), a result which would be consistent with ganetespib induction of premature senescence.

**Figure 4:**
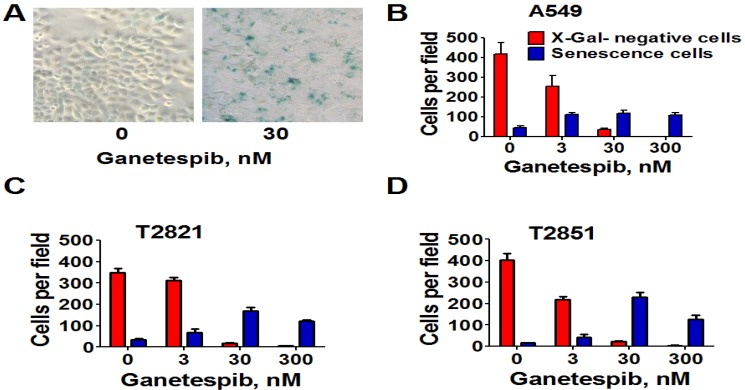
Senescence-associated β-galactosidase staining of ganetespib treated lung AC cells. Cells were treated with ganetespib (0–300 nM) for 3 days and senescence-associated β-galactosidase (SA-β-gal) staining was performed according to manufacturer instructions. Senescent cells were identified as cells stained blue. (**A**). Representative images (light microscope, 10 × magnification) of A549 cells that were stained for SA-β-gal after treatment with ganetespib (0, 30 nM), for 72 h are presented. (**B**)–(**D**). The numbers of SA-β-gal β-positive cells and negative cells in cultures after the treatment with ganetespib (0, 3, 30 and 300 nM) are shown.

Next, we analyzed for senescence-associated expression of β-galactosidase (SA-β-gal). As shown in Figure 4, the numbers of SA-β-gal-Positive cells in all cell lines were significantly higher when treated with 3 nM ganetespib than in the untreated control cultures. Furthermore as the ganetespib dosage was increased greater of numbers of SA-β-gal positive cells were detected. Overall, these data suggests that ganetespib produces its antitumor effect by multiple mechanisms in lung adenocarcinoma cells; including apoptosis, irreversible growth arrest and premature senescence.

### 2.4. Ganetespib Increases the Radiosensitivity of Lung Adenocarcinoma Cells in Vitro

**Figure 5:**
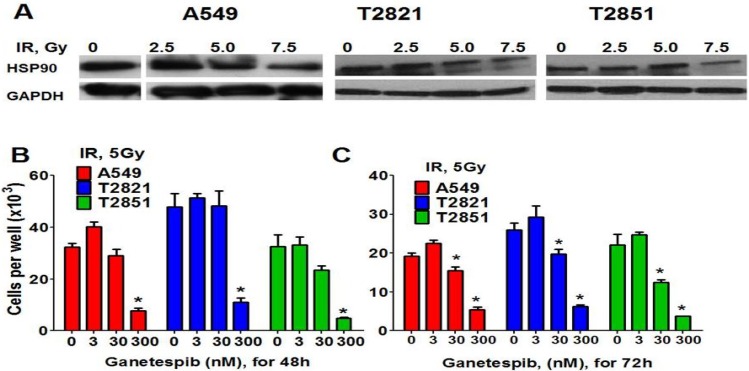
HSP90 is expressed in IR-treated cells. HSP90 inhibitor ganetespib sensitizes cells to IR. (**A**) Western blots analysis of HSP90 expression in IR-treated AC cells. Cells were treated with IR, (0–7.5 Gy). At 72 h post-radiation, lysates were prepared and immunoblotted using antibodies against HSP90. GAPDH was included as a control. (**B**), (**C**) Ganetespib sensitizes lung AC cells to IR. The cells were treated with the combination of IR (5Gy) with ganetespib (0, 3, 30, 300 nM) and were stained with Hoechst33342 and counted at 48 h and 72 h post-radiation. Results are expressed as the mean ± SD of three independent experiments.

Western blot analysis showed that HSP90 was abundantly expressed in IR-treated cells (Figure 5A), raising the possibility that combining ganetespib with IR might increase the efficacy of antitumor therapies. A combined treatment regime utilizing ganetespib (0–300 nM) with cell irradiation (5 Gy) was tested for its effects on cell proliferation (Figure 5B,C). The combination of IR and 300 nM ganetespib dramatically reduced cell numbers at 48 and 72 h while the radiosensitizing effect of 30 nM ganetespib was only observed 72 h post-treatment. This combined treatment resulted in an increase in the number of cells undergoing mitotic catastrophe as well as cell death via apoptosis (data not shown). Low dose treatment of cells with ganetespib at 3 nM had no detectible effect (Figure 5B,C). Next the “classical” clonogenic survival assay was employed to test whether pretreatment with ganetespib and also post radiation treatment has similar radiosensitizing effects in NSCLC cells. Previously it was shown that the pretreatment of cells with HSP90 inhibitors led to radiosensitizing effect in different cancer cells [61,62,63,64].

**Figure 6:**
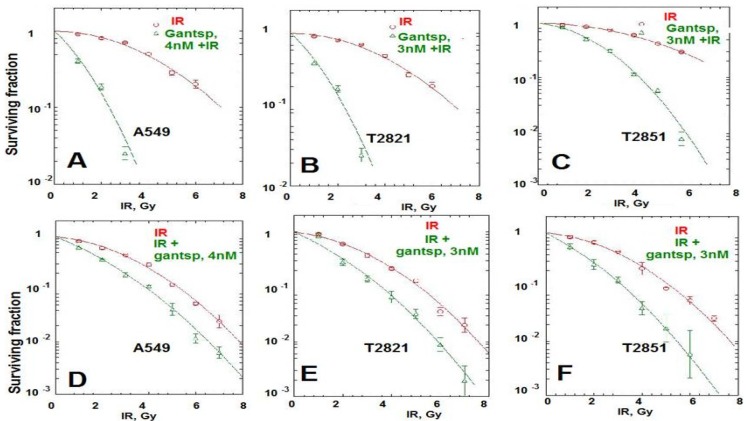
Pretreatment or post-radiation treatment of lung AC cells with ganetespib at a low concentration significantly reduces clonogenic survival. (**A**)–(**C**). Pretreatment AC with ganetespib radiosensitizes AC cells to IR. Cells were plated. The next day, ganetespib (3 nM or 4 nM) was added into the culture media for 24 h; then adherent cell cultures were irradiated (0–10 Gy). Cells were fixed and clonogenic survival was estimated on the seventh day after IR treatment. Radiation survival curves showing IR-sensitivity for cell lines, A549 (**A**), T2821 (**B**) and T2851(**C**) that were pretreated with ganetespib (3 nM or 4 nM, green line) and without pretreatment (red line). (**D**)–(**F**). Post-radiation treatment of lung AC cells with ganetespib significantly reduces clonogenic survival. Cells were suspended, irradiated (0–10 Gy) and plated. The next day, ganetespib was added into the culture media. Cells were fixed and clonogenic survival was estimated on the seventh day after IR treatment. Radiation survival curves show IR-sensitivity of A549 (**D**), T2821 (**E**) and T2851 (**F**) cells grown without drug (red line), or grown in the presence of ganetespib (3 nM or 4 nM, green line). Average data of five experiments are shown.

Here we found that pretreatment of AC cells with ganetespib at low nanomolar concentrations (3 nM and 4 nM) for 24 h before IR treatment dramatically increased the radiosensitivity of AC cells (Figure 6A–C). Importantly, if ganetespib at the same low concentrations has been added into the culture media 24 h after IR, it also dramatically augment the antitumor effects of IR in decreasing the survival rate of IR-treated cells (Figure 6D–F and Table 1). A significant reduction in the proliferating fraction of T2821 and T2851 cells was shown following the treatment of cells with IR in combination with ganetespib, 3 nM, (Figure 6A,C,D), with a dose enhancement ratio of 1.36 and 1.47, respectively (Table 1). Ganetespib (4 nM) also effectively sensitized A549 cells to IR (Figure 6A,B; Table 1). Taken together, this data demonstrates that both pretreatment with ganetespib and post radiation treatment with ganetespib has significant radiosensitizing effects in lung AC cells harboring diverse oncogenic mutations such as KRAS and EGFR.

**Table 1:** Comparison of radiosensitivity of NSCLC cells treated with HSP90 inhibitor ganetespib after irradiation.

Cell Line	T2821		T2851		A549	
Treatment	IR	IR + ganetespib, 3 nM	IR	IR + ganetespib, 3 nM	IR	IR + ganetespib, 4 nM
D_0_	1.279 ± 0.12	* 1.047 ± 0.03	1.351 ± 0.07	* 1.28 ± 0.02	1.511 ± 0.07	* 1.358 ± 0.02
ñ	5.314 ± 0.91	* 3.018 ± 0.26	4.901 ± 0.34	* 1.360 ± 0.34	3.830 ± 0.76	* 1.677 ± 0.15

Results were calculated with linear quadratic and single hit multi-target models and presented as D_0_-Gy, (the dose required to reduce the fraction of survived cells to 37%) and ñ (extrapolation number derived by extension of the linear proportion of the IR survival curve to the ordinate of the graph using a log/linear plot). * *p* < 0.05 (compared to IR group).

### 2.5. Ganetespib Inhibits Repair of IR-Induced DNA Damages in NSCLC Cells

**Figure 7:**
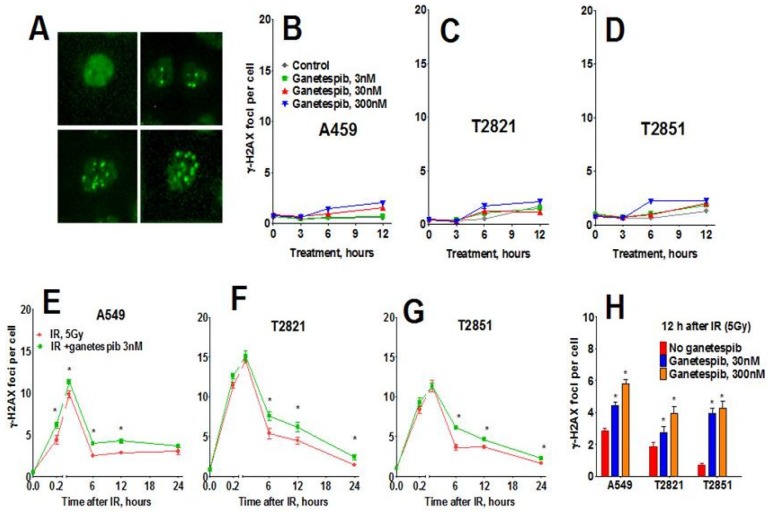
Effect of ganetespib on IR-induced γH2AX foci formation and foci duration. Cells were pretreated with ganetespib (0, 3, 30, 300 nM) for 2 h, irradiated (5 Gy) and cultured for 0–24. The cells were stained for γH2AX and with Hoechst 33342 and analyzed using HCA/HCS methods. (**A**). The representative images of control and irradiated cells stained for γH2AX foci are shown. (**B**)–(**D**). Ganetespib as monotherapy has a negligible effect on γH2AX formation. (**E**)–(**G)**. Ganetespib at a low concentration of 3 nM influenced IR-induced γH2AX foci formation. Average numbers of the γH2AX foci per cell in AC cells treated with ganetespib in combination with IR, 5 Gy are shown at different time post-radiation. (**H**). Combining IR with ganetespib at higher concentrations (30, 300 nM) inhibits DNA damage repair and γH2AX foci resolving. Average numbers of the γH2AX foci per cell are presented. Each point represents average data for 3000 cells. Data are expressed as the mean ± SD of three experiments.

Next, we assessed the phosphorylated histone H2AX (γH2AX) foci, a sensitive indicator of DNA damage in the form of DNA double strand breaks (DSB). The γH2AX foci could be visualized as a bright spot that was present in all IR-treated cells (Figure 7A). No difference in γH2AX foci was observed at any time in AC cells exposed to either 3 or 30 nM ganetespib as a monotherapy (Figure 7B–D). However, treatment with 300 nM ganetespib did influence the formation of the γH2AX foci, suggesting that high concentration of drug could augment DNA damage. Ganetespib did not influence γH2AX foci formation at 0.2–1 h post-radiation. Six hour post-radiation, the numbers of γH2AX foci were reduced by approximately 1/3 of the level detected at 1 h, suggesting that by this time most DNA repair had already occurred. The differences between IR and IR treatment combined with ganetespib appeared 6–24 h post-radiation (Figure 7). Level of γH2AX foci significantly higher in cells treated with IR + ganetespib compared to cells treated with IR only (Figure 7). This delayed dispersal suggests that ganetespib inhibits the repair of DSBs induced by IR and increases radiosensitivity.

**Figure 8:**
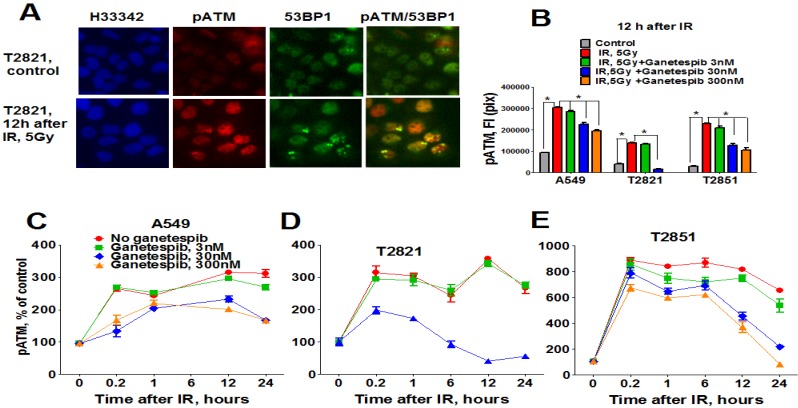
Ganetespib downregulates ATM phosphorylation in IR-treated lung AC cells. A549, T2821 and T2851 cells were pretreated with ganetespib (0–300 nM) for 2 h, irradiated (5 Gy) and grown for 0–24 h before fixation. The cells were stained for pATM, 53BP1 and with Hoechst 33342. Cell images were analyzed using HCA/HCS methods. (**A**). Images of T2821 cells stained for pATM, and 53BP1 are shown. (**B**)–(**E**). Ganetespib affects pATM expression in irradiated cells. The total average fluorescence intensities of pATM in cells were determined at 12 h post-radiation (**B**). Time-dependent and concentration dependent effect of ganetespib on the levels of pATM present in irradiated A549 (**C**), T2821 (**D**) and T2851 (**E**) cells are presented. Data are shown as % of the control.

In light of these findings, we investigated the status of key DNA repair proteins—pATM (ataxia telangiectasia mutated), ATR (ataxia telangiectasia and Rad3-related), and 53-binding protein 1 (53BP1) in lung AC cells treated with the combined treatment of IR plus ganetespib. As expected, IR upregulated pATM expression; with the highest degree of pATM upregulation found in T2851 cells where it co-localized with γH2AX and 53BP1 foci (Figure 8). Pretreatment of cells with ganetespib decreased pATM levels in IR-treated cells in all three cell lines (Figure 8B–D).

**Figure 9:**
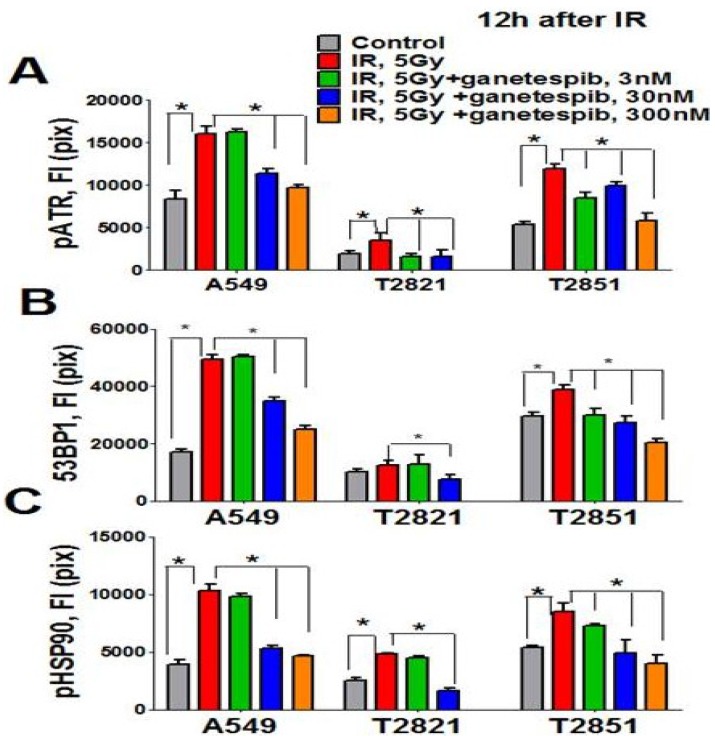
Ganetespib inhibits 53BP1, phospho-ATR and phospho-HSP90 nuclear proteins expression in IR-treated lung AC cells. Cells were pretreated with ganetespib (0–300 nM) for 2 h, irradiated (5 Gy) and grown for 12 h. The cells were stained for pATR (Ser473), 53BP1, and pHSP90 (Th-7) and with Hoechst 33342. Cell images were analyzed using HCA/HCS methods. Ganetespib downregulated pATR (**A**), 53BP1 (**B**), and pHSP90 (**C**) proteins expression in IR-treated cells. The total average fluorescence intensities of proteins in control cells and cells treated with IR alone or in combination with increasing concentrations of ganetespib are shown.

Furthermore, activation of both ATR and 53BP1 in irradiated AC cells was also affected by ganetespib treatment (Figure 9A,B). Taken together, these data suggest that treatment with nanomolar concentrations of ganetespib impacts multiple proteins involved in the DNA damage repair process.

The direct involvement of HSP90 in the DNA damage response has been recently demonstrated [65]. It was found that the Thr-7 residue of HSP90 was phosphorylated immediately after DNA damage, and that pHSP90 accumulated at sites of DNA DSBs [65]. To determine whether HSP90 is directly involved in the DNA repair response in IR treated lung AC cells, the level of pHSP90 (Th-7) proteins present was assayed using the HCS and HCA methods. Irradiation of cells increased detected levels of pHSP90 proteins. The pHSP90 proteins were mainly located in the nuclei and co-localized with γH2AX foci in IR-treated lung AC cells. The lung AC cells which received the combined treatment showed a significant down regulation of pHSP90 proteins (Figure 9C). Interestingly, a difference was detected in the levels of protein expression (pHSP90, pATM, pATR and 53BP1) in the different cell lines. T2821 showed reduced protein expression as compared to the T2851 and A549 cells (Figure 8 and Figure 9). Our data suggests that the combined ganetespib and IR treatment impacted several overlapping pathways involved in DNA repair and might lead to the formation of persistent DNA damage foci.

### 2.6. Ganetespib at 3 nM Concentration Influences Cell Signaling Activity in Irradiated AC Cells

PI3K/AKT signaling is activated in response to IR treatment in NSCLC cells [66]. Ganetespib (10–1000 nM) has been shown to inhibit PI3K/AKT signaling in NSCLC cells [48,49,50,67].

**Figure 10:**
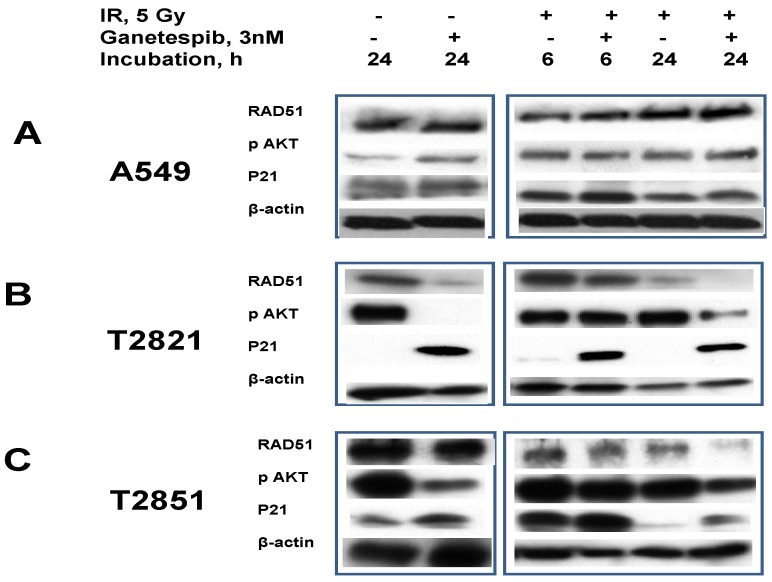
Ganetespib, 3 nM, inhibits IR-induced AKT phosphorylation, degrades the DNA repair protein RAD51 and induces p21 accumulation. **A**–**C**: A549, T2821 and T2851 cells were irradiated (0, 5 Gy), and incubated with ganetespib (0, 3 nM) for 6 or 24 h. Cell lysates were prepared and immunoblotted using antibodies against RAD51, pAKT, and p21. β-actin was included as a control.

Here we examined the modulation of PI3K/AKT signaling in irradiated A549, T2821 and T22851 cells pretreated with ganetespib, 3 nM, using western blot (Figure 10). pAKT was highly expressed in untreated cells indicating the important role of PI3K/AKT signaling in primary T2821 and T2851 cells. Ganetespib, 3 nM, completely inhibited AKT phosphorylation in T2821 cells, and significantly suppressed the p-AKT level in T2851 cells; however ganetespib, 3 nM, have no effect in A549 cells (Figure 10). IR dramatically elevated the level of p-AKT in AC cells, however, post -radiation treatment with ganetespib significantly suppressed p-AKT levels in T2821 and T2851 cells and did not affect level of p-AKT in A549 cells (Figure 10).

RAD51, a major protein involved in homologous recombination (HR), is involved in the repair of replication-induced DSBs and DSBs induced by IR [19,68,69]. RAD51 is missing in DNA-SCARs that are formatted in radiation-induced senescence cells [70], and the RAD51 expression in tumor cells might be affected by HSP90 inhibitors [71].

To investigate these observations, we tested the effect of ganetespib, 3 nM, on RAD51 expression in irradiated lung AC cells (Figure 10). RAD51 was found to be abundantly expressed in naïve AC cells. In IR treated cells the RAD51 levels were found to be upregulated in only the T2821 cells. Following the post-radiation treatment of cells with ganetespib (3 nM) RAD51 protein were found to be degraded in T2821 and T2851 primary cell lines, but not affected in A549 cells (Figure 10).

In contrast to RAD51, the CDK inhibitor p21 is present at very low levels in naïve AC cells and ganetespib, 3 nM, increased expression of p21 in AC cells (Figure 10). Notably, the combination of IR with ganetespib treatment dramatically upregulated the level of p21 in cells, suggesting that ganetespib might potentiate IR-initiated damage resulting in induced growth arrest and senescence.

### 2.7. Combining Ganetespib with IR Increases Level of Persistent DNA Damages Triggering Senescence

IR may cause unrepaired DSBs leading a persistent DNA damage response and to the generation of persistent foci and DNA-SCARS that functionally regulate multiple aspects of the senescent phenotype. DNA-SCARS lack RAD51 and require pATM, 53BP1 and γH2AX as important DNA-SCARS stabilizing components [70,72].

**Figure 11:**
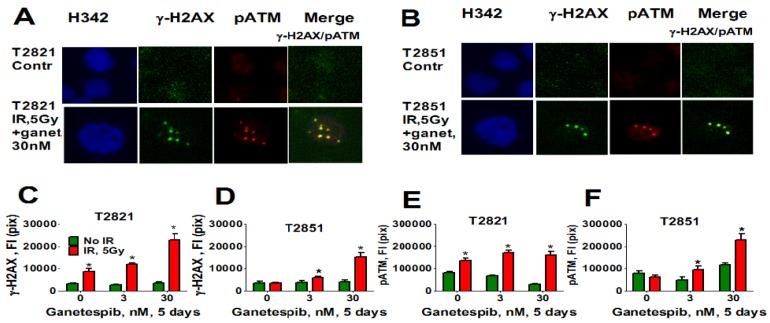
Combining IR with ganetespib increases the level of the persistent DNA damage foci while accumulating DDR mediators (pATM and γH2AX). T2821 and T2851 cells were irradiated (0, 5 Gy), and cultured in the presence of ganetespib (0–30 nM) for 5 days. The cells were stained for pATM (Ser1981), γH2AX and with Hoechst 33342 and were analyzed using HCA/HCS methods. (**A**), (**B**). Co-localization of pATM (red) and γH2AX (green) in persistent IR-induced foci. The representative images of T2821 cells (**A**) and T2851 cells (**B**), untreated cells and cells which were irradiated and grown with ganetespib (30 nM) for 5 days, are shown. (**C**)–(**F**). Ganetespib triggers the accumulation IR-induced persistent DNA damage foci. The total average fluorescence intensities of γH2AX and pATM in control cells and cells treated with IR alone or in combination with increasing concentrations of ganetespib were determined at 5 days post-radiation.

**Figure 12:**
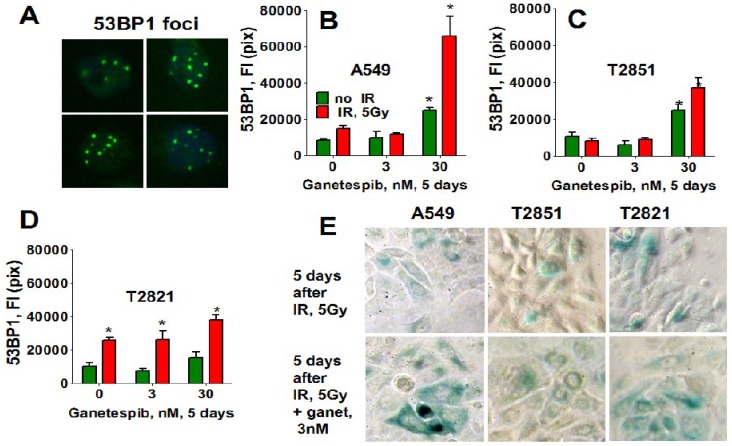
Ganetespib upregulates the level of 53BP1 in IR-induced persistent DNA damage foci. A549, T2821 and T2851 cells were irradiated (0, 5 Gy), and cultured in the presence of ganetespib (0–30 nM) for 5 days. The cells were stained for 53BP1 and were analyzed using HCA/HCS methods. (**A**). The representative images of T2821 cells stained for 53BP1 are shown. (**B**)–(**D**). The total average fluorescence intensities of 53BP1, γH2AX and pATM in control cells and cells treated with IR alone or in combination with increasing concentrations of ganetespib were determined at 5 days post-radiation. (**E**). Senescence-associated β-galactosidase staining of AC cells treated with IR (5 Gy) and the combination of IR (5 Gy) with ganetespib (3 nM) at 5 days post-radiation. Representative images (light microscope, 10× magnifications) of cells stained for SA-β-gal are shown. Senescent cells were identified as cells stained blue. *, significance of differences between ganetespib treated cells and nontreated cells at *p* < 0.05

Here, we analyzed the effect of ganetespib at 5 days post-radiation on the expression of pATM, γH2AX (Figure 11), 53BP1 (Figure 12A–D) and SA-β-gal (Figure 12E) in AC cells. γH2AX and pATM proteins were found to be co-localized in persistent foci (Figure 11A). Only irradiated T2821 cells demonstrated an upregulation of pATM and γH2AX proteins; however, a dramatic upregulation of pATM and γH2AX was observed in all AC cell lines treated with the combined treatment of IR and ganetespib (Figure 11, shown for T2851 and T2821 cells).

Similarly, only T2821 cells showed an upregulation of 53BP1 expression after IR treatment (Figure 12), while 53BP1 protein expression was elevated in all AC cells treated with the combined treatment with IR and 30 nM ganetespib. T2821 cells which received the combination IR and a low dosage of ganetespib, 3 nM, also demonstrated upregulation of 53BP1 protein expression (Figure 12). These findings indicate that, depending upon the cellular context, ganetespib may enhance the formation of IR-induced persistent DNA damage foci, and cellular senescence.

### 2.8. Ganetespib Inhibits T2821 Xenografted Tumors Growths and Potentiates Effect of IR Treatment

**Figure 13:**
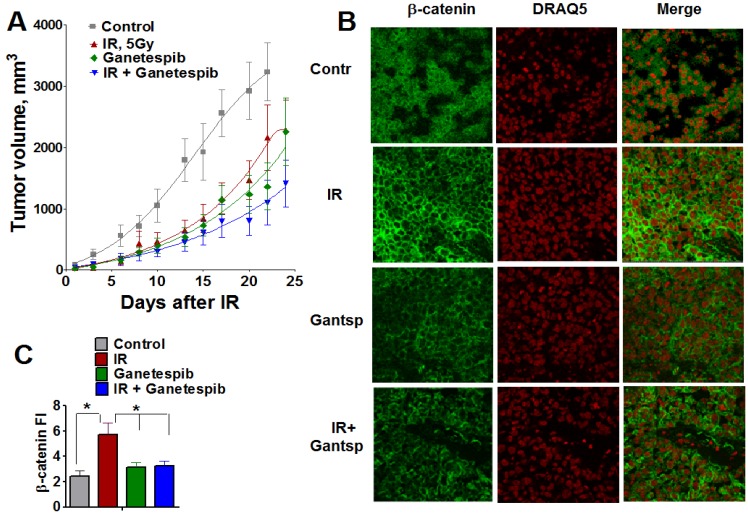
Combined treatment IR with ganetespib effectively inhibits the growth of T2821 tumors xenografted in mice. Mice bearing established T2821 tumors in the right hind limbs were pretreated with ganetespib and only the tumor region was irradiated with a single dose of 5 Gy. Ganetespib (25 mg/kg) was administered twice a week. Tumors were measured every 2-3 days, and tumor volumes were calculated. (**A**): Analysis of the tumor growth. (**B** and **C**): Xenografted tumor irradiation leads to dramatic upregulation of β-catenin expression in tumor tissues. The combination of IR and ganetespib mitigates the β-catenin upregulation in T2821 tumor tissue. *, significance of differences between groups of tumor tissues at *p* < 0.05.

Finally, the antitumor effects of IR, ganetespib and the combination of IR with ganetespib treatments were assessed in a T2821 human lung AC xenograft model (Figure 13, Table 2). Our results show that all three treatments were tolerable for animals. Treatments of xenografted tumors with a single dose of IR (5 Gy), or with ganetespib applied as a monotherapy at a low concentration of 25 mg/kg twice a week, resulted in significant and similar levels of tumor growth reduction (Figure 13A).

The statistical analysis of the individual tumor growth data revealed that by combining IR treatment with ganetespib resulted in a significant inhibition in tumor growth as compared to tumor growth in mice that received the IR only or ganetespib only treatments (Figure 13A, Table 2). 

**Table 2:** Comparison of T2821 tumors growth curves.

Comparison	*p*-value for the Comparison of Tumor Growth Slope Using *F*-test in the Mixed Model	Adjusted *p* Value with the Bonferroni Correction
IR *vs.* untreated	0.0062	0.0372
Ganetespib *vs.* untreated	<0.0001	<0.001
IR + Ganetespib *vs.* untreated	<0.0001	<0.001
Ganetespib *vs.* IR	0.0814	0.4884
IR + ganetespib *vs.* IR	<0.0001	<0.001
IR + ganetespib *vs.* ganetespib	<0.0001	<0.001

This finding demonstrates that the HSP90 inhibitor, ganetespib, significantly sensitizes T2821 lung AC cells to IR treatment *in vivo*.

### 2.9. IR Treatment of The Tumors Upregulates β-catenin Expression; However, Combining IR with Ganetespib Suppresses β-catenin Level in T2821 Xenografted Tumors

Wnt/β-catenin is involved in the regulation of stemness, EMT and metastasis in lung cancer [55,73,74]. To verify whether the radiosensitizing effect of ganetespib is associated with an alteration in β-catenin expression in lung tumors, we employed immunofluorescent staining for β-catenin in T2821 tumor tissue sections assessed by confocal microscopy (Figure 13B,C). Ganetespib exposure alone did not affect β-catenin expression in tumor tissues; whereas, IR-monotreatment of the tumors led to a dramatic upregulation of β-catenin expression in tumor tissues; however, by combining ganetespib treatment with IR significantly decreased the level of β-catenin in T2821 xenografts (Figure 13B,C). This result suggests that combining IR with HSP90 inhibition may mitigate the activation of WNT/beta-catenin signaling in IR treated cells.

### 2.10. Discussion

In this research, we have demonstrated for the first time that ganetespib treatment results in significant radiosensitization of lung AC cells *in vitro* and *in vivo*. Ganetespib has been shown to have this effect on AC cells of differing genetic backgrounds including KRAS mutant A549 cells, EGFR mutant primary T2851 cells, and primary T2821 cells that express wt KRAS and EGFR. Our data shows a strong cytotoxic effect for ganetespib as a mono-therapeutic in treatment of AC cells, and that ganetespib effectively sensitized AC cells to IR in combined treatments. Pretreatment of AC with ganetespib at low nanomolar concentrations (3 nM or 4 nM) for 24 h before gamma-irradiation dramatically increased the efficacy of IR treatment *in vitro*. These findings are in concordance with previous observations of the radiosensitizing effects of different HSP90 inhibitors in various cancer cells lines [61,62,63,64], our findings suggest that ganetespib is the most effective radiosensitizing SP90 inhibitor in lung AC cells.

Of particular significance is our finding that post-radiation treatment of AC cells with ganetespib (3, 4 nM) also could potentiate antitumor effect of ionizing radiation, as we have shown using clonogenic survival assay *in vitro*. Further research is needed to elucidate the mechanisms of the observed post radiation activity of ganetespib.

Of even greater importance were our results for xenografted tumors grown *in vivo* and treated with ganetespib. In these experiments we found that ganetespib inhibited the T2821 xenografted tumors growth and potentiated the antitumor effect of IR. Our study suggests that the ganetespib radiosensitization effects are probably actuated through multiple mechanisms as the HSP90 protein has many protein clients.

HSP90 inhibitors, as radiosensitizing agents, have significant promise because the molecular chaperone, HSP90, supports the many molecular pathways driving tumorigenesis along with IR therapy resistance [34,35,51]. The inhibition of HSP90 results in the degradation of its client proteins, leading to the concomitant disruption of multiple oncogenic signaling cascades through one molecular target [33,35,75]. Since the initial discovery of HSP90 as a target for anticancer therapy, incredible progress has been made in developing potent first- and second-generation HSP90 inhibitors. HSP90 inhibitors could sensitize a variety of tumor cell lines to IR [61,62,63,64]. However, there are no HSP90 inhibitors currently approved for cancer therapy [76].

Ganetespib, a new second-generation inhibitor of HSP90, showed activity in NSCLC both as monotherapy and in combination with docetaxel [35,47]. Our findings complement and extend those of the recent studies, showing that ganetespib as monotherapy exerts robust antitumor activity in different types of cancer [6,46,49,77,78].

In the previous studies, the most common responses of the cells to HSP90 inhibition were cell cycle arrest, which can occur through G1/M or G2/M transition arrest and it is likely dependent of CDK4, CDC2 and polo-like kinases [46,47,49,67,79]. We found that the treatment of lung AC cells with ganetespib alone, at low a nanomolar concentration, may lead to multiple antitumor effects such as: Inhibition of cells migration; cell proliferation and cell cycle progression leading to accumulation of cells in G2/M; activation of programmed cell death by apoptosis; and activation of the premature senescence. Ganetespib, as a single agent, inhibits the expression of antiapoptotic proteins (AKT and pAKT) in lung AC cells. This is the same for other cancer types [67,77,80,81,82], suggesting that ganetespib may translate well as a single agent for lung cancer treatment.

Using γ-H2AX foci as a marker for DSB, we determined that ganetespib, at the concentrations of 3 and 30 nM, did not induce DSBs while ganetespib at a higher concentration of 300 nM might induce DNA damages. Notably, the treatment of AC cells with ganetespib, at a low concentration of 3 nM, led to the degradation of RAD51 which is one of the major proteins involved in HR [19,68,69].

An important finding of this study was the capacity of ganetespib to induce permanent growth arrest and potentiate IR-induced senescence in lung AC cells. This is a new and significant feature of this drug because the stimulation of cellular senescence has been considered as a novel approach to improve cancer therapy with less severe effects than cytotoxic therapy [83]. Unlike apoptotic cells, which rapidly disintegrate, senescence cells remain viable for long intervals and are metabolically active, but are permanently in growth arrest. Several signaling cascades are associated with the establishment of senescence phenotype: Classical pathways such as p53 which activates p21; and p16 that prevents phosphorylation of the retinoblastoma protein (Rb) [70,84,85,86]. Another well-known senescent pathway is PTEN-p27. P27 protein (with inhibitory activity on CDK2-cyclinE) is frequently down regulated in many cancers and it’s expression is restored in senescence cells [87]. We showed that AC cells treated with ganetespib for 3–5 days display the key defining features of premature senescence cells: They are metabolically active, express markers of a senescence state such as p21, p53, and p27 and they are SA-β-gal positive. T2821 and T2851 cells treated with ganetespib, at a nanomolar concentration for 24 h, led to the degradation of the RAD51 DNA repair protein, downregulation of AKT and pAKT; upregulation of p21 and accumulation of persistent, non-reparable DNA damage foci. The p16-Rb pathway is not associated with senescence in AC cells treated with ganetespib, either with monotherapy or in combination with IR treatment. Other researchers have found similar results in their studies of another HSP90 inhibitor in nasopharyngeal carcinoma cells [88].

High migration and invasion capacities are key characteristics of metastatic tumor cells and lung cancer stem-like cells (CSCs) [28,89,90,91]. To date, drugs capable of blocking metastasis are not available [57]. Our findings that the HSP90 inhibitor ganetespib, at a nanomolar concentrations, effectively reduces the migration of AC cells, suggests an important anti-metastatic activity for ganetespib. Anti-migratory activity of other HSP90 inhibitors have also demonstrated that inhibition of HSP90 can function in blocking tumor cell migrations [92,93].

Now, it is generally accepted that the IR treatment of tumor cells *in vitro* may select for CSCs and induces EMT [28,94,95,96,97]; and the IR-resistance of these cells is associated with the activation of the Wnt/β-catenin signaling pathway in different cancers [98,99,100]. Here, using T2821 xenografted tumor tissue samples from our *in vivo* study, we have determined that a single fraction of IR leads to the significant upregulation of β-catenin expression in T2821 xenografted tumors. However, beta-catenin had no nuclear, but membrane or outside of nucleus localization. By combining IR with ganetespib treatment mitigates β-catenin upregulation, keeping concentrations of β-catenin similar to that seen in untreated tumors. This research is very promising and needs further investigation.

Our observation that ganetespib in nanomolar concentrations dramatically inhibits the growth of lung tumor spheres in stem cell under selective conditions, suggests that ganetespib is able to abrogate radiation induced selection for lung CSCs and raises the possibility that the HSP90 inhibitor, ganetespib, is not only a potent radiosensitizing drug but also an antimetastatic agent.

We determined that treatment with ganetespib induced radiosensitization in lung AC cells *in vitro* and *in vivo*. Our study suggests that ganetespib may impart radiosensitization through multiple mechanisms: (1) re-assortment of lung AC cancer cells into G2-M; (2) induction of apoptosis and mitotic catastrophe; (3) down regulation of the PI3K/Akt radioresistance pathway; (4) delay of DNA repair after IR treatment; and (5) accelerate IR-induced senescence.

In response to IR induced DNA damages, cells typically activate ATM-CHK2 and ATR-CHK1 signaling pathways to arrest the cell cycle and initiate DNA repair [21]. For these processes phosphorylation of H2AX (γ-H2AX) is necessary for the recruitment of many factors involved in DNA repair [101,102]. ATM, ATR, and DNA-PKcs are members of the phosphatidylinositol-3 kinase-like kinase (PIKK) family; and PIKKs are dependent on HSP90 for their activation via the R2TP complex and Tel2 [103]. HSP90 inhibition with ganetespib impacts multiple signaling cascades, including the activation of ATM, ATR and other PIKKs in IR-treated lung AC cells. This leads to the delay of DNA repair and to the accumulation of unrepaired, persistent γ-H2AX foci which has been discovered in senescence cells. Recent reports have shown the ability of HSP90 inhibitors to prolong the duration of γ-H2AX in irradiated prostate [64], breast, and cervical cancer cell lines [104]. However, we are the first to recognize the significant impact of HSP90 inhibition on the formation of the senescence phenotype in IR-treated lung AC cells.

Previous reports suggest that the HSP90 inhibitors radiosensitize the human prostate [105] and head and neck squamous cell carcinoma [106] cells by the abrogation of RAD51-mediated HR. Our data demonstrates the ganetespib-driven loss of RAD51, in both irradiated and non-irradiated cells, indicating that the radiosensitizing effect of ganetespib is likely associated not only with the degradation of RAD51 but with the diminishing of multiple important DNA repair pathways and downregulation of important DNA repair proteins (such as ATM, ATR, 53BP1, *etc.*). Our data also shows an upregulation and nucleus localization of pHSP90 in irradiated AC cells, suggesting that HSP90 is directly involved in the DNA repair processes; nevertheless, this finding needs further investigation. In addition, our data suggests that the radiosensitizing effect of HSP90 inhibitors may arise from the broad down-regulatory effects (direct or indirect) of multiple critical radioresistance pathways, whose components are clients of the HSP90 [61,107].

An important finding of this study is the capacity of ganetespib to significantly improve the efficacy of radiation therapy in T2821 xenografts. Our data indicates a clear clinical relevance for combining ganetespib with radiation in the treatment of lung AC.

## 3. Experimental Section

### 3.1. Cells

Human A549 cells were purchased from the American Type Culture Collection (ATCC, Manassas, VA, USA). Cells were grown in culture media, as per ATCC recommendations, with supplemental 10% FBS (Atlanta Biologicals, GA, USA). Surgical lung adenocarcinoma samples were obtained from the University of Pittsburgh Cancer Institute (UPCI) Lung Specialized Program of Research Excellence (SPORE) with written consent from patients under an approved Institution Review Board protocol by University of Pittsburgh Scientific Review Committee (IRB# 01-09-27-07). Patient anonymous samples were used in the experiments according to protocol. We isolated T2821 and T2851 lung AC cells from surgical tumor samples (Table 3). To generate a stock of these cells, they were expanded in culture prior to harvesting and cryopreservation. While under investigation, T2821 and T2851 cells were grown in RPMI media supplemented with 10% FBS.

**Table 3:** NSCLC sources for primary adenocarcinoma cell cultures.

Tumor ID	Tumor Stage	Primary Tumor ?	Mutations			EML4/ALK
			KRAS	BRAF	EGFR	
T2821	IV( T4Nx)	No. Second, T2N1	No	No	No	No
T2851	T2N0	Yes	No	No	Yes, exon 21	No

### 3.2. Reagents

Hoechst 33342, anti-β-actin, anti-GAPDH, and anti-HMGB1 antibodies were purchased from Sigma-Aldrich (Sigma-Aldrich, St. Louis, MO, USA). Draq5^TM^ was purchased from eBioscience (San Diego, CA, USA). Antibodies against p16^INK4a^ and pATM (Ser1981) were purchased from Abcam Inc. (Abcam, Cambridge, MA, USA). p21^WAF1/Cip1A^, ATM, AKT, pAKT (Ser 473), p ATR (Ser428) and β-catenin antibodies were purchased from Cell Signaling Technology Inc., (Danvers, MA, USA). Antibodies against 53BP1 and γH2AX (Ser139) were obtained from EMD Millipore (Darmstadt, Germany). Rad51 and ATR antibodies were obtained from Santa Cruz Biotechnology, (Dallas, TX, USA). Antibodies against p53 and p62 were acquired from Novus Biologicals (Littleton, CO, USA). The phalloidin-FITC and secondary antibody conjugated with Alexa^®^-488, -546, and -680 were obtained from Molecular Probes (Invitrogen, Carlsbad, CA, USA). The HSP90 inhibitor ganetespib [3-(2,4-dihydroxy-5-isopropylphenyl)-4-(1-methyl-1*H*-indol-5-yl)-1*H*-1,2,4-triazol-5(4*H*)-one] was synthesized and kindly provided by Synta Pharmaceuticals Corp. (SYNTA Pharmaceuticals, Lexington, MA, USA).

### 3.3. Irradiation

AC cells were irradiated *in vitro*, as cell suspension or as monolayer, using the Shepherd Mark 168 Irradiator, (^137^Cs Irradiator) (JL Shepherd, San Fernando, CA, USA) at a dose rate of 70.6 cGy per hour at room temperature. For our *in vivo* experiments, we used a Varian linear accelerator using 6 MeV photons with a dose rate of 300 monitor units/min (Varian Medical Systems, Inc., Palo Alto, CA, USA). The mice received a single dose of IR according to published methods [108]. The region of the right hind limbs of the mice (region that is bearing xenografted T2821 tumor cells) was irradiated with a single dose of 5 Gy. The animal body was shielded by a 10/_2_ mm value layer block so that only the right hind limb region was irradiated [108].

### 3.4. Cells Staining Procedure for Cellomics Array Scan Automated Imaging

Cells were fluorescently stained as described [28,55]. Cells grown in 96-well plates and were fixed in 2% PFA for 20 min, permeabilazed with 0.1% Triton X-100 for 10 min, and incubated with primary antibodies for 1 h and with secondary antibodies for 1 h. Cell nuclei were then stained with Hoechst 33342 at 2 μg/mL for 20 min to identify individual cells and to optimize focusing. All incubation and fixation procedures were performed at room temperature. Cell images were acquired using the Cellomics ArrayScan HCS Reader (Cellomics/ThermoFisher, Pittsburgh, PA, USA) and were analyzed using the Compartment or Target Activation BioApplication Software Modules. Data was captured, extracted and analyzed with ArrayScan II Data Acquisition and Data Viewer version 3.0 (Cellomics).

### 3.5. Cell Proliferation and Viability Assays

AC cells were seeded in triplicate in 96 well plates and incubated for 24–72 h in the absence or presence of different concentrations of ganetespib (0–200 nM). The number of viable cells was determined using 3-4,5-dimethyldiazol-2-yl]-2,5-diphenyltetrazolium bromide (MTT, Sigma-Aldrich) conversion assay, according to the manufacture’s protocol. In some experiments, cells were counted using the Cellomics ArrayScan HCS Reader after staining with 2 μg/mL of DNA binding dye, Hoechst 33342, for 20 min.

### 3.6. Tumor Sphere Growth

Suspension growth of AC cells was assessed as described [28,55]. Single cell suspensions were prepared and plated at 100–500 cells/mL in ultra-low attachment 24 well plates (Corning, Corning, NY, USA) in MammoCult™, which is a serum-free liquid culture medium (Stem Cell Technologies, Vancouver, BC, Canada). Ganetespib (0–20 nM) was added into culture medium, and cells were incubated for 10 days. The tumor sphere growth was analyzed under a phase-contrast microscope with 10× objective and counted from at least three wells.

### 3.7. In Vitro Clonogenic Assays

To analyze effect of the pretreatment with ganetespib on clonogenic survival of irradiated cells, exponentially growing cells were harvested by exposure to trypsin and plated in 6 well plates, 500 cells per well. The next day, ganetespib (3 nM or 4 nM) was added into the culture media for 24 h; then adherent cell cultures were irradiated using a 137Cs gamma-ray (0–10 Gy). Cultures were incubated at 37 °C in 5% CO_2_. Cells were fixed and stained with crystal violet; colonies ≥ 50 cells were counted and the size of the colonies were measured (Gel Count colony counter, Oxford Optronix, Oxford, UK) as we described [28].

To analyze effect of the post-radiation treatment with ganetespib, cell suspensions were prepared and irradiated with doses ranging from 0 to 10 Gy and plated in 6 well plates, 500 cells per well. Cultures were incubated at 37 °C in 5% CO_2_. The next day, HSP90 inhibitor ganetespib was added to the cultures at the final concentrations of 0–10 nM. At the seventh day after irradiation, cells were fixed stained with crystal violet; and counted as we described [28].

### 3.8. Immunofluorescence Detection of γH2AX, pATR, pATM, pHSP90 and 53BP1

Cells growing in 96 well plates were pretreated with ganetespib (0, 3, 30, 300 nM) for 2 h, then the cells were irradiated 0–5 Gy at RT. After IR treatment, cells were incubated in the culture media supplemented with 10% of FBS, 37 °C, 5% CO_2_ for 0–30 h. Then the cells were fixed, permeabilazed and incubated with antibodies against γH2AX, 53BP1, p-ATR, p-ATM and p-HSP90 at room temperature for 1 h.

### 3.9. Apoptosis Assay

Apoptosis was analyzed by flow cytometry using Alexa Fluor 488-conjugated AnnexinV and propidium iodide (PI) (Molecular Probes, Eugene, OR, USA) as recommended by the manufactures. In this assay Cells were first treated with ganetespib (0, 3, 30, 300 nM) for 0, 6, 24 or 48 h then harvested, washed twice with ice cold PBS, resuspended in a 500 μL incubation buffer containing Annexin V-Alexa Fluor 488 and propidium iodide (PI), and incubated in the dark for 15 min. Analyses were performed using a Accuri C6 Flow Cytometer (Becton Dickinson, Franklin Lakes, NJ, USA).

### 3.10. Cell Cycle Analysis

Cells growing in 6 well plates were exposed to the indicated concentrations of ganetespib (0, 30 nM) for 48 h. Cells were then harvested and resuspended in ice cold PBS. Iced cold 70% ethanol was used to fix the cells and they were stored overnight at 4 °C, washed twice with PBS, and resuspended in 50 μg/mL PI staining reagent containing 100 μg/mL RNase and 0.1% Triton X-100 for 30 min in the dark. Cells were analyzed by flow cytometry (Accury C6, Becton-Dickinson) and the percentage of cells in the different phases of the cell cycle was analyzed with Becton-Dickinson software.

### 3.11. Senescence and Autophagy Assays

Senescence-associated β-galactosidase (SA-β-gal) activation was detected in ganetespib treated cells as described [109]. Cells growing in 24-well plates were treated with ganetespib for 0–5 days, and stained with X-Gal (Cell Signaling Inc., Senescence β-Galactosidase Staining Kit) according to the manufactures protocol. The senescent SA-β-gal + cells (stained blue) were determined by bright-field microscopy. Cells images were captured and numbers of senescent cells and unstained cells were counted in 5–10 fields in each well. Other senescence-associated markers, cyclin-dependent kinase inhibitors, p16^INK4a^, p21^WAF1/Cip1^, and p53, as well as autophagy associated markers, sequestosome-1 (p62), light chain 3 (LC31/2) and High mobility group box 1 (HMGB1) were evaluated in ganetespib-treated cells using the Cellomics ArrayScan as described above.

### 3.12. Monolayer Wound Healing Assay

Cells were seeded into six-well plates at high density and were cultured for 2 days to produce monolayers. Confluent cell monolayers were then scratched using a plastic 10 µL-pipette tip as described [28]. Wounded monolayers were then washed four times with medium to remove cellular debris and incubated in culture medium supplemented with 10% FBS for 24 h. Images were captured at 0, 2, 4, 6 and 24 h post treatment using a ZEISS (light) microscope Axiovert 40C (Hamamatsu, Japan). Resulting wound diameters were measured.

### 3.13. Western Blotting

Western blotting analysis was performed as previously described [110]. The tumor cells were treated, and harvested at different time points. The harvested cells were disrupted using lysis buffer (Cell Signaling Technology, Danvers, MA, USA) in the presence of protease inhibitor and incubated on ice for 30 min (vortex every 10 min). Lysates were clarified by centrifugation and equal amounts of proteins resolved by SDS-PAGE before transfer to PVDF membrane (Bio Rad, Hercules, CA, USA). The membranes were blocked with 5% non-fat milk, incubated with primary antibodies followed by addition of secondary antibodies. Chemiluminescence signals were detected on the X-ray film. As an internal control the GAPDH or β-actin primary antibody was also probed. The gel-digiting software Un-Scan-It gel (Version 5.1; Silk Scientific, Inc., Orem, UT, USA) was used to quantify the intensities of Chemiluminescent bands detected.

### 3.14. Tumor Xenografts

Six- to eight-week-old female NOG-F mice, (Taconic Labs, Hudson, NY, USA) were housed according to IACUC protocols at the Hillman Cancer Center—University of Pittsburgh Cancer Institute. All protocols were approved by the IACUC of the University of Pittsburgh. Veterinary care was provided by the Division of Laboratory Animal Research of the University of Pittsburgh. To establish tumor xenografts, T2821 human adenocarcinoma cells were *s.c.* inoculated into the right hind limbs of NOG-F mice (2 × 10^6^ cells per mouse) as described [111]. Mice bearing established tumors (100–200 mm^3^) were randomized into treatment groups: (1) Control; (2) IR; (3) HSP90 inhibitor ganetespib; (4) IR + Hsp90 inhibitor. The region of the right hind limb was irradiated with a single dose of 5 Gy according to published methods [108]. Mice received an intra-peritoneal (*i.p.*) injection of either ganetespib (25 mg/kg) formulated in 10/18 DRD [10% dimethyl sulfoxide (DMSO), 18% Cremophor RH 40, 3.6% dextrose, 68.4% water] or 10/18 DRD without ganetespid 48 h prior to IR treatment. Ganetespib (25 mg/kg) treatments were continued twice a week for the duration of the experiment. Tumor volumes (mm^3^) were defined as follows: [(W_1_ × W_1_ × W_2_) × (π/6)], where W_1_ and W_2_ were the smallest and the largest tumor diameters (mm), respectively. Tumor growth inhibition was determined as described previously [112].

### 3.15. Tumor Analysis

Tumor tissue samples from all treatment groups were embedded in OCT Compound (Miles), snap frozen, and stored at −80 °C. Cryostat sections (8 μm) were stained using hematoxilin and eosin and histopathologically evaluated. Three slides from each tumor were incubated with monoclonal anti-beta catenin antibody and cell nuclei were stained with Draq5^TM^. Slide images were acquired with Leica Laser Confocal Microscope, model DMRE TCS SL, and analyzed using ImageJ software.

### 3.16. Statistics

IR survival curves for *in vitro* treatment groups were analyzed by comparison with the linear-quadratic and the single-hit multi-target models. Statisical comparisons used the final slope of the survivorship curves, representing multiple-event killing (D_0_), and an extrapolation of the width of the shoulders on the radiation survival curve (ñ) [113]. Results for D_0_ and ñ were summarized as the mean ± the standard error (SEM) from multiple measurements and were compared using a two-sided, two-sample *t*-tests. In other *in vitro* experiments, comparisons between groups was done with the two-sided two-sample *t*-tests if the data was normally distributed; or Wilcoxon rank sum tests if not. One-way or two-way ANOVA were used for comparing multiple groups, followed by two sample *t*-tests for pair wise comparisons. For all these statistical analyses, a *p*-value of 0.05 was regarded as significant.

Individual xenografted tumor growth curves were plotted by treatment group (untreated, IR, ganetespib, and IR + ganetespib). The tumor volume data were log-transformed, and a linear mixed model was built on the log-transformed volume data, where treatment, time of tumor measurement in days, and treatment by time interaction were used as fixed effects, and we assumed a random intercept and a random slope for each mouse. The Mixed procedure (*i.e.*, Proc Mixed) in SAS (SAS Institute, Cary, NC, USA) was used to fit this model. The comparison of slopes between any two groups was performed in this model with an *F*-test, using Contrast statement in Proc Mixed. The *p*-values were adjusted with the Bonferroni correction for multiple comparisons.

## 4. Conclusions

Our study demonstrated that the HSP90 inhibitor, ganetespib, has robust antitumor activity as both a monotherapy in lung AC cells along with its significant effect in potentiation of ionizing radiation *in vitro*, in combined therapy with ionizing radiation. We determined that lung adenocarcinoma cells harboring diverse oncogenic mutations such as KRAS and EGFR were sensitized to ionizing radiation. Ganetespib was also shown to: Inhibit cell growth and migration, tumor sphere formation induced apoptosis and cell cycle redistribution, led to irreversible cell growth arrest and the development of the senescence phenotype in lung adenocarcinoma cells. The most clinically significant finding of this study is our demonstration that treatment with ganetespib has the capacity to significantly improve the efficacy of IR therapy in primary human lung adenocarcinoma T2821 xenografts.
